# Cytotoxicity and Gene Expression Changes of a Novel Homeopathic Antiseptic Oral Rinse in Comparison to Chlorhexidine in Gingival Fibroblasts

**DOI:** 10.3390/ma13143190

**Published:** 2020-07-17

**Authors:** Masako Fujioka-Kobayashi, Benoit Schaller, Michael A. Pikos, Anton Sculean, Richard J. Miron

**Affiliations:** 1 Department of Cranio-Maxillofacial Surgery, Inselspital, Bern University Hospital, University of Bern, 3010 Bern, Switzerland; masako.kobayashi@insel.ch (M.F.-K.); benoit.schaller@insel.ch (B.S.); 2 Private Practice, Pikos Institute, Trinity, FL 34655, USA; mapikos@gmail.com; 3 Department of Periodontology, University of Bern, 3010 Bern, Switzerland; anton.sculean@zmk.unibe.ch

**Keywords:** chlorhexidine, anti-septic solution, cell viability, periodontal disease, oral rinses

## Abstract

Most available antiseptic solutions available today have strong antibacterial effects, however most also possess major cytotoxic effects on human gingival tissues. The VEGA Oral Care Recovery Kit (StellaLife), previously evaluated in clinical studies, consists of 16 active ingredients that are monographed in the Homeopathic Pharmacopeia of United States (HPUS) and recognized for their accelerated healing properties (reduction in post-op pain). The aim of this study was to compare VEGA to chlorhexidine (CHX) in vitro on gingival fibroblast viability, survival at various concentrations, migration assay, proliferation activity, expression of both regenerative growth factors as well as inflammatory markers, and collagen synthesis. A 10-fold dilution of standard CHX (0.02%) led to cell death, whereas cell viability was significantly better in the VEGA group for all tested parameters. Furthermore, VEGA also induced significantly greater fibroblast migration and proliferation. CHX negatively impacted the cellular inflammatory response of gingival fibroblasts, and also led to a reduction in collagen synthesis (50% decrease). Findings from the present study provide support from basic laboratory experiments that validate the previous clinical studies supporting the use of the VEGA oral rinse on its superior biocompatibility and wound healing properties when compared to CHX.

## 1. Introduction

In the United States, opioids are commonly prescribed by dentists in oral surgery for the management of acute pain, but their use has been put into question in recent years based on the number of teenage children who have reported their drug abuse/addictions to other narcotics often began with opioids prescribed by healthcare providers. Since the mid-1990s, deaths caused specifically from opioid overdose has more than quadrupled, which parallels precisely the increase in opioid prescriptions done in dental and medical offices [1,2].

For these reasons, alternative strategies have been proposed in recent years with the aim of lowering postoperative pain following dental therapy. Recently, a novel, opioid-free VEGA oral care recovery kit has been recognized as an effective treatment to reduce pain owing to its more natural formulation. It consists of 16 active ingredients that are monographed in the Homeopathic Pharmacopeia of United States (HPUS) and recognized for their accelerated healing properties with antimicrobial properties [3]. Lee and Suzuki utilized the VEGA oral care recovery kit to evaluate pain, wound healing, edema, and ecchymosis in two groups—one with opioid use and the other without—in patients who underwent harvesting of monocortical blocks of bone from the posterior mandible and ramus with local anesthesia [3]. The authors reported that this novel, opioid-free alternative form of therapy administered preemptively prior to block bone graft surgery resulted in decreased postoperative pain and less reliance of opioid analgesics [3]. Furthermore, a study conducted by Tatch also found a 3-fold reduction in prescribed opioids when the VEGA oral care recovery kit was added to routine private practice procedures over a 3-year period (>1000 patients evaluated—reduction in opioids prescribed from 59% down to 19% of patients) [4].

It is reported by the developer that a single application of VEGA Oral Rinse is reported to significantly reduce anaerobic bacterial numbers including *Streptococcus mutans*, Actinomyces viscosus, *Streptococcus pyogenes*, *Porphyromonas gingivitis*, and *Bacteroides fragilis*. Nevertheless, no data to date has investigated its effect at the cellular level on gingival fibroblasts or other cell types found in the oral cavity. Therefore, the aim of the present study was to compare the effects of the VEGA oral care recovery solution to the commonly utilized pre-/postoperative agent chlorhexidine (CHX). The effects on human gingival fibroblast viability, survival at various concentrations, migration assay, proliferation activity, expression of platelet-derived growth factor-a (PDGF-a) and inflammatory markers including tumor necrosis factor-α (TNF-α) and interleukin (IL)-6, and collagen synthesis were investigated in vitro.

## 2. Materials and Methods 

### 2.1. Reagents and Cells

Two antiseptic oral rinses—VEGA^®^ Oral Care (StellaLife, Northbrook, IL, USA) and chlorhexidine gluconate (CHX, 0.2%, Dentohexine, Uznach, Switzerland)—were tested in the present study. The original concentration of the products was considered 100% throughout the study. VEGA oral rinse contains 0.55% each of the following HPUS ingredients: Azadirachta indica (1×), Calendula (1×), Echinacea purpurea (1×), Plantago major (1×).

Human gingival fibroblasts (HGF-1) were used in the present study (ATCC, Manassas, VA, USA). HGF-1 cells were cultured in a humidified atmosphere at 37 °C in DMEM (Gibco, Thermo Fisher Scientific, Waltham, MA, USA), with 10% fetal bovine serum (FBS; Gibco) and 1% antibiotics (Gibco). Cells were detached from tissue culture plastic by using 0.25% EDTA-Trypsin (Gibco) prior to reaching confluency. 

### 2.2. Cell Viability Assay

HGF-1 cells were seeded at a density of 25,000 cells on 8-well chamber slides (Nunc Lab-Tek II Chamber Slide, Nunc, Thermo Fisher Scientific). At 24 h post-seeding, cells were exposed to both antiseptic solutions for 10 s, 1 min, 3 min, or 5 min, then rinsed in PBS twice. The treated cells were then visually evaluated for cell viability using a live–dead assay (Enzo Life Sciences AG; Lausen, Switzerland). According to the manufacturer, a cell-permeable green fluorescent dye (Ex(max): 488 nm; Em(max): 518 nm) is utilized to stain live cells. Dead cells are stained by propidium iodide (PI) and a red fluorescent dye (Ex(max): 488 nm; Em(max): 615 nm), which in viable cells is actively pumped out of the cytoplasm. Stained cells are visualized by fluorescence microscopy using a band-pass filter (detects FITC and rhodamine). Fluorescent images were captured with a fluorescent microscope (Nikon Eclipse E800, Nikon, Tokyo, Japan). 

Cell viability of HGF-1 cells were further quantified when cultured in diluted antiseptic oral rinses (0.1%, 1%, 10%, or 100%) up to 240 min by a luminescent cell viability assay (CellTiter-Glo^®^, Promega, Madison, WI, USA). HGF-1 cells were seeded at a density of 5000 cells per well in 96-well plates. After 24 h, the diluted antiseptic oral rinses (0.1%, 1%, 10%, or 100%) were exposed to cell culture media. At desired time points (10 s, 1 min, 10 min, 60 min, and 240 min), media was changes in flesh culture media and living cells were quantified by a luminescence plate reader (TECAN Infinite200 pro, Tecan Group Ltd., Männedorf, Switzerland).

### 2.3. Cell Migration Assay

The 24-well plate and polyethylene terephthalate filters with a pore size of 8 μm (ThinCertTM, Greiner Bio-One GmhH, Frickenhausen, Germany) were used for the cell migration assay. Ten-thousand cells starved in DMEM containing 0.5% FBS for 12 h were seeded in the upper compartment in DMEM containing 0.5% FBS. The DMEM containing 10% FBS and 1% antiseptic oral rinses were filled into the lower compartment of the wells. For control samples, the regular DMEM culture media was filled in lower wells. After 24 h, cells were fixed in 4% formaldehyde for 2 min, followed by permeabilized with methanol (Sigma, St. Louis, MO, USA) for 15 min, and then stained with Giemsa (MERCK, Darmstadt, Germany) for 20 min. The upper side of the filter membrane was rinsed in water and gently wiped by a cotton swab to remove the cell debris. The numbers of cells on the lower side of the filter were counted by the counting tool of the digital microscope (Keyence, Osaka, Japan).

### 2.4. Proliferation Assay

HGF-1 cells were seeded at a density of 2500 cells per well in 96-well plates. After 24 h, the media was changed into 1% of each antiseptic oral rinse in cell culture media. Cell proliferation was quantified by a luminescent cell viability assay (CellTiter-Glo^®^, Promega, Madison, WI, USA) at 1, 3, and 5 days. 

### 2.5. Real-Time PCR Analysis

Cells were seeded at a density of 50,000 cells for real-time PCR per well in 24-well plates. After 24 h, the media was replaced into 1% of each antiseptic oral rinse in cell culture media. Total RNA was isolated from HGF-1 cells using a ReliaPrep™ RNA Cell Miniprep System (Promega, Madison, WI, USA), at 5 days post-stimulation of antiseptic oral rinses to investigate the mRNA expressions of collagen 1a2 (COL1), platelet-derived growth factor-a (PDGF-a), tumor necrosis factor-α (TNF-α), and interleukin (IL)-6. Primer sequences were shown in Table 1. Real-time RT-PCR was performed using the GoScript™ Reverse Transcription system (Promega, Madison, WI, USA), and quantified on an Applied Biosystems 7500 fast machine with using GoTaq^®^ qPCR Master Mix (Promega, Madison, WI, USA). The ∆∆Ct method was utilized to calculate gene expression levels normalized to the expression of glyceraldehyde 3-phosphate dehydrogenase (GAPDH) and β-Actin.

### 2.6. Collagen Immunofluorescent Staining

HGF-1 cells were seeded at a density of 25,000 cells per well in 8-well chamber slides (Nunc). At 24 h, cells were treated in 1% antiseptic oral rinses (either VEGA or CHX) on chamber slides. At 7 days post-stimulation, HGF-1 cells were fixed with 4% formaldehyde for 10 min, followed by permeabilized cells in 0.2% Triton X-100 in PBS for 15 min, and blocked in 1% bovine serum albumin (BSA, Sigma) in PBS for 1 h. Thereafter, the cells were incubated overnight at 4 °C with mouse monoclonal collagen type I (COL-1) antibody (dilution 1:100, sc-59772, Santa Cruz, Dallas, TX, USA). FITC-conjugated mouse IgG binding proteins (dilution 1:100, sc-516140, Santa Cruz) were used for 1 h for visualization. The specimens were then mounted with Vectashield containing DAPI (Vector, Burlingame, CA, USA). Images were captured with a Nikon fluorescence microscope. The optical density of the collagen staining was quantified using ImageJ software (NIH, Bethesda, MD, USA).

### 2.7. Statistical Analysis

Means and standard errors were presented, and the data were analyzed for statistical significance using a one-way analysis of variance for migration assay and collagen staining, and two-way analysis for the cell viability and proliferation assay with the Bonferroni test of variance, and the nonparametric Kruskal–Wallis test for real-time PCR analysis (*p*-values < 0.05 were considered significant) by GraphPad Prism 8.3 software (GraphPad Software, Inc., La Jolla, CA, USA).

## 3. Results

### 3.1. Cytotoxity of VEGA and CHX on Human Gingival Fibroblasts

**Figure 1:**
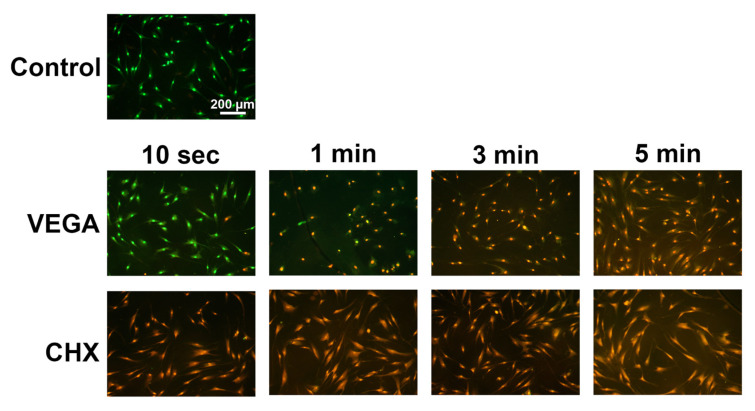
Cell viability of human gingival fibroblasts (HGF-1 cells) exposed to 100% of antiseptic oral rinses (either VEGA or CHX) for 10 s, 1 min, 3 min, or 5 min, respectively. Live–dead staining was done with viable cell appearing in green and red cells stained with PI representing dead cells. VEGA showed viable cells at 10 s and 1 min treatments, whereas cells cultured with CHX demonstrated dead cells in as little as 10 s.

The effects of two antiseptic oral rinses were investigated on the cell viability of human gingival fibroblasts (HGF-1 cells). Not surprisingly, it was first observed that 100% of either antiseptic oral rinses (0.2% CHX or 0.55% active ingredients in VEGA) resulted in high levels of PI staining (representing cell death) within 3 min after exposure (Figure 1). Interestingly, all earlier time points including rinses for 10 and 1 min demonstrated more viable cells in the VEGA group (green cells) when compared to CHX (even at 10 s) (Figure 1).

**Figure 2:**
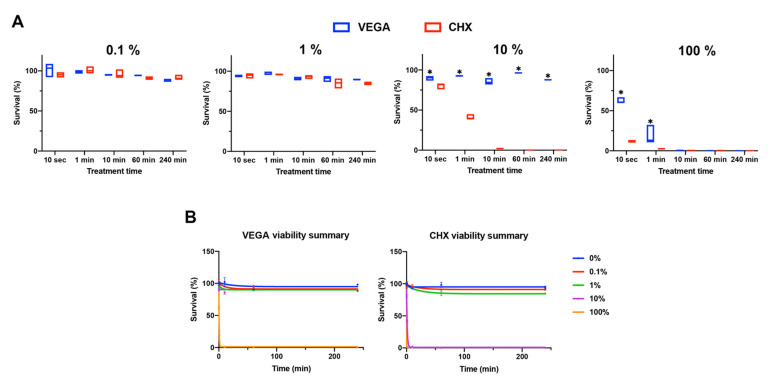
Cell viability of HGF-1 cells exposed to 0.1%, 1%, 10%, or 100% of antiseptic oral rinses (either VEGA or CHX) for 10 s, 1 min, 10 min, 60 min, and 240 min. (**A**) Two antiseptic oral rinses at each dilution were compared for cell survival properties. (n = 3, * denotes significantly higher than the other treatment modality, *p* < 0.05). (**B**) The effect of dilutions of antiseptic oral rinses on cell survival was summarized. In general, 0.1% and 1% antiseptic oral rinses did not affect cell viability.

Thereafter, diluted versions of either antiseptic oral rinse were investigated for cell survival up to 240 min (Figure 2). VEGA demonstrated significantly higher cell survival than CHX when treated in 10% and 1% initial concentrations (i.e., 0.02% CHX and 0.002% CHX, respectively) (Figure 2A). Most notably, at 10%, by 1 min more than 50% cell death was observed in the CHX group, and by 10 minutes, 100% of cells were no longer viable. In contrast, cells exposed to 10% VEGA demonstrated 100% cell survival up to 240 min (Figure 2A). As cell survival remained high for both antiseptic oral rinses at a 1% concentration, the 1% dilution was therefore chosen for further cell assays. 

### 3.2. Influence of VEGA and CHX on Human Gingival Fibroblast Migration and Proliferation

**Figure 3:**
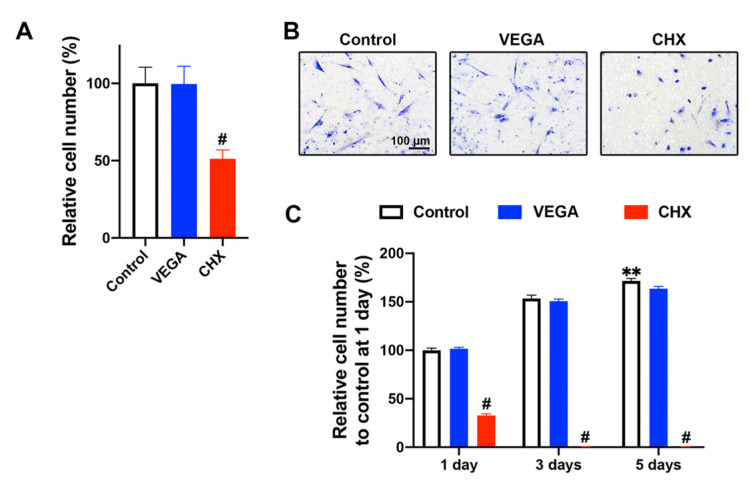
The effects of antiseptic oral rinses on HGF-1 (**A**,**B**) cell migration and (**C**) proliferation. (**A**) The migrated cell numbers (n = 9) and (**B**) images of the migrated cells at 24 h after either 1% VEGA or CHX treatment. (**C**) Proliferation assay of HGF-1 cells exposed to either 1% of VEGA or CHX at 1, 3, and 5 days (n = 5). It was found that 1% VEGA treatment showed similar cell migration and proliferation properties to control, whereas 1% CHX treatment inhibited cell migration and proliferation (** denotes significantly higher than all other treatment modalities. * denotes significant difference between groups. # denotes significantly lower than other modalities, *p* < 0.05).

VEGA and CHX were then investigated on HGF-1 cells for the potential of cell migration and proliferation (Figure 3). The 1% of the initial CHX concentration inhibited cell migration by ~50% when compared to control (Figure 3A). In contrast, cells cultured with 1% VEGA did not negatively impact the cell migration (Figure 3A,B). In line with these findings, a proliferation assay demonstrated that CHX treatment significantly decreased cell numbers at 1, 3, and 5 days when compared to those of the control and VEGA groups (Figure 3C).

### 3.3. Influence of VEGA and CHX on Human Gingival Fibroblast Gene Expressions and Collagen Synthesis

**Figure 4:**
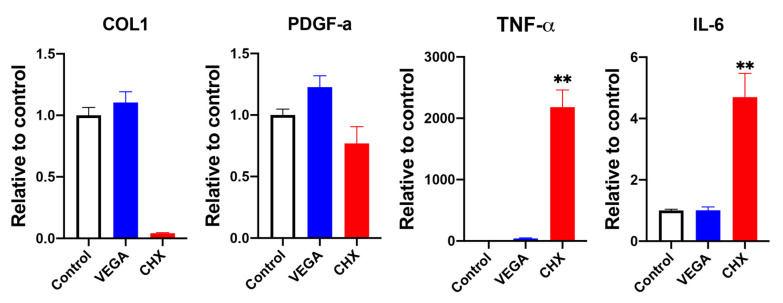
Real-time PCR of HGF-1 cells cultured in either 1% VEGA or CHX at 5 days displaying mRNA levels of COL1, PDGF-a, TNF-α, and IL-6 (n = 3, ** denotes significantly higher than all other treatment modalities, *p* < 0.05).

Investigation into mRNA levels revealed that CHX decreased COL1 and PDGF expression and increased inflammatory cytokines expression, including TNF-α and IL-6, when compared to expressions in the VEGA treatment (Figure 4). Noteworthy, while collagen gene expression in the VEGA group was similar when compared to controls, a drastically reduction in the CHX group was observed (Figure 4).

**Figure 5:**
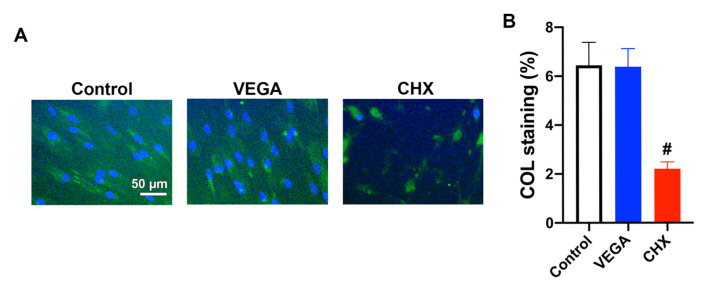
Immunofluorescent collagen 1 (COL1) staining of HGF-1 cells treated in 1% of either VEGA or CHX at 7 days. (**A**) The merged fluorescent images of COL1 staining (green) with DAPI staining (blue). (**B**) Quantified values of COL staining (n = 9, # denotes significantly lower than other modalities, *p* < 0.05).

Similarly, while VEGA did not induce any chance in the inflammatory response of gingival fibroblasts when compared to control cells, a marked and significant increase in inflammatory markers was observed in the CHX group. Similarly, while collagen synthesis was maintained in the VEGA group when compared to controls, over a 3-fold decrease in collagen production was observed in the CHX group following 7 days of cell culture (Figure 5). 

## 4. Discussion

Antiseptic solutions are routinely used in the dento-maxillofacial field for a variety of procedures including pre- and postoperative rinses [5], conservative treatment for osteonecrosis of the jaw [6,7], relieving treatment for oral mucositis [8], irrigation of extraction sockets [9,10], in the field of periodontology as antibacterial mouth rinses [11], irrigation of dental implants [12,13], and for the treatment and management of advanced cases of periodontitis [14,15,16]. Based on their widespread use, it becomes pivotal that their effect on soft and hard tissue healing is uneventful and that their interface with cells of the oral cavity is favorable.

To date, the majority of research on antiseptic solutions has focused on bacterial growth inhibition, but data on their impact directly on normal mesenchymal and eukaryotic cells from host body has been sparse in comparison. Previous reports have in fact shown that commonly utilized antiseptic solutions may have a negative role on epithelial cells [17], fibroblasts [18], and gingival cells [19]. Previously, our research team investigated four commonly utilized antiseptic solutions, including (1) povidone-iodine (PI, 0.5%), (2) CHX (0.2%), (3) hydrogen peroxide (H_2_O_2_, 1%), and (4) sodium hypochlorite (HYP, 0.25%) on bone chip cell viability and growth factor release of VEGF, TGFB1, BMP2, RANKL, and IL1β [20]. It was actually reported following antiseptic rinsing that the CHX-rinsed bone chips demonstrated the highest cellular viability between all the antiseptic solutions however nearly 50% of the cells underwent dead [20,21].

Based on our group’s previous results, it remained of interest to further investigate the findings based on the study by Lee and Suzuki utilizing a novel VEGA oral care recovery kit favoring pain scores, wound healing, edema, and ecchymosis [3]. In that study, thirty-four patients were randomly divided into two groups: Group A was provided the opioid-free VEGA regimen pre/post-surgery and Group B was provided standard opioid analgesics. Following surgery, the primary outcomes measured was pain intensity measured, on a 10 point space. Strikingly, the pain scores from their study showed a decrease in pain score (5.6 versus 7.1) when patients were placed on the VEGA oral care recovery program when compared to test opioid patients [3]. The authors concluded that “this novel, opioid-free alternative form of therapy administered preemptively prior to block bone graft surgery resulted in decreased postoperative pain, less reliance of opioid analgesics, and increased the time interval towards the use of a rescue opioid analgesic”. The authors further made the remark that they believe that there is a definite role in novel opioid-free alternative therapy in managing postoperative pain [3].

Based on these findings, we investigated for the first time the effects of VEGA on gingival fibroblast cell activity. Interestingly, it was first observed that the biocompatibility was far superior when compared to CHX, specifically at dilutions of 1% and 10% original clinically recommended values (0.2% CHX and 0.55% active ingredients in VEGA). Noteworthy, standard in vitro protocols typically utilize ~100-fold dilutions for in vitro cell studies when compared to actual clinical concentrations [22,23]. Two important findings were noted in this study: (1) the CHX group failed to demonstrate any ability for cells to migrate/proliferate over time (Figure 2 and Figure 3) and (2) actually further caused a dramatic 3-fold increase in the inflammatory marker IL-6 and over a 100-fold increase in TNF-α (Figure 4). Therefore, one can only question if selecting CHX as a pre-/post-op oral rinse may actually be causing a delay in wound healing, an increase in inflammation, and subsequent pain leading to patients desiring opioids. While this hypothesis certainly requires clinical evaluation, the data combined with previous clinical studies certain eludes to this possibility.

Previously, a study by Fadia et al. concluded that CHX induced apoptosis of cultured fibroblasts at lower concentrations and necrosis at higher concentrations, and increased expression of the heat-shock protein 70, an indicator of cellular stress [24]. In an original article dating back more than 20 years, Mariotti and Rumft [25] warned the dental community that their results on CHX suggested that it induced a dose-dependent reduction in cellular proliferation and that even concentrations of CHX that have little effect on cellular proliferation can significantly reduce both collagen and non-collagen protein production of human gingival fibroblasts in vitro. They further suggested that the introduction of commercially available concentrations (0.12%) or diluted commercial concentrations (as low as 0.00009%) of CHX to surgical sites for short periods of time prior to wound closure can conceivably have serious toxic effects on gingival fibroblasts and may negatively affect wound healing [25]. We confirm these previous findings and further demonstrate that CHX cut the production of collagen in half when exposed to a concentration diluted 100-fold when compared to their current uses in clinical practice and further induced over a 100-fold increase in certain proinflammatory genes (Figure 5).

In conclusion, the findings from the present study report that the use of VEGA oral rinse was far superior to CHX in terms of cellular biocompatibility, ability to induce cell migration, proliferation, gene expression, and collagen synthesis. We further demonstrated the negative impact of CHX on the cellular inflammatory response of human gingival fibroblasts that may further lead to reported patient pain. Last, we provide support from the basic laboratory that further validates the previous clinical studies supporting the use of the VEGA oral rinse on its superior biocompatibility when compared to CHX.

## 5. Conclusions

The findings from the present study report that the use of VEGA oral rinse was far superior to CHX in terms of cellular biocompatibility, ability to induce cell migration, proliferation, gene expression, and collagen synthesis. We further demonstrated the negative impact of CHX on the cellular inflammatory response of human gingival fibroblasts that may further lead to reported patient pain. Last, we provide support from the basic laboratory that further validates the previous clinical studies supporting the use of the VEGA oral rinse on its superior biocompatibility when compared to CHX.

## Figures and Tables

**Table 1:** Primers for quantitative RT-PCR.

Gene	Primer Sequence (5′–3′)	
	Forward	Reverse
hCOL1	CCCAGCCAAGAACTGGTATAGG	GGCTGCCAGCATTGATAGTTTC
hPDGF-A	CACACCTCCTCGCTGTAGTATTTA	GTTATCGGTGTAAATGTCATCCAA
hTNF-α	CAGCCTCTTCTCCTTCCTGAT	GCCAGAGGGCTGATTAGAGA
hIL-6	GAAAGGAGACATGTAACAAGAGT	GATTTTCACCAGGCAAGTCT
hGAPDH	AGCCACATCGCTCAGACA	GCCCAATACGACCAAATCC
hβ-Actin	CCAACCGCGAGAAGATGA	CCAGAGGCGTACAGGGATAG

hCOL1 = human collagen 1, hPDGF-A = human platelet derived growth factor-A, hTNF-α = human tissue necrosis factor-alpha, hIL-6 = human interleukin-6, hGAPDH = human glyceraldehyde 3-phosphate, hβ-Actin = human Beta-actin.
